# Rescue under ongoing CPR from an upper floor: evaluation of three different evacuation routes and mechanical and manual chest compressions: a manikin trial

**DOI:** 10.1186/s13049-020-0709-0

**Published:** 2020-03-04

**Authors:** Hendrik Drinhaus, Sebastian Nüsgen, Niels Adams, Wolfgang A. Wetsch, Thorsten Annecke

**Affiliations:** 1 Department of Anaesthesiology and Intensive Care Medicine, University of Cologne, Faculty of Medicine and University Hospital of Cologne, Kerpener Str. 62, 50937 Cologne, Germany; 2 Fire Brigade, Brühl, North Rhine-Westphalia Germany

**Keywords:** Out-of-hospital cardiac arrest, Cardiopulmonary resuscitation, Emergency medical services, Transport under ongoing cardiopulmonary resuscitation, Fire service, Mechanical chest compressions

## Abstract

**Background:**

If transport under ongoing cardiopulmonary resuscitation (CPR) from an upper floor is indicated, the ideal CPR-method and evacuation route is unknown hitherto. We aimed to elaborate a strategy for evacuation of patients under ongoing CPR from an upper floor, comparing three different evacuation routes and manual and mechanical chest compressions.

**Methods:**

A CPR-training manikin recording CPR-quality was placed on the fifth floor and was evacuated to an ambulance via lift, turntable ladder, or staircase. Chest compressions were performed manually or with a mechanical CPR-device. Efficiency endpoints were compression depth and frequency, sufficiency of chest release, compared with European Resuscitation Council (ERC) Guidelines, and duration of the evacuation. Adverse outcomes were disconnection/dislocation of devices and hazards/accidents to the personnel.

**Results:**

For all evacuation routes, compression depth and frequency were significantly more compliant with ERC-guidelines under mechanical CPR. Manual CPR was associated with considerable deviations from correct compression depth and frequency. Chest release only slightly differed between groups. Evacuation via lift under mechanical CPR was fastest and evacuation via turntable ladder under manual CPR was slowest. No device disconnections or accidents occurred, but hazard to personnel was perceived during evacuation via ladder under manual CPR.

**Conclusions:**

In this study, a mechanical CPR-device proved to deliver better CPR-quality during evacuation from an upper floor. If a lift accessible with a stretcher is available, this route should be preferred, regardless of manual or mechanical CPR. Turntable ladders can only be meaningfully used with mechanical CPR, otherwise CPR-quality is poor and hazard to the personnel is increased. Not all evacuation routes may be useable in a specific real-life scenario.

**Trial registration:**

German Clinical Trials Registry, www.drks.de, registration number DRKS00012885, registration date 17.08.2017.

## Background

Out-of-hospital cardiac arrest (OHCA) is usually treated by performing cardiopulmonary resuscitation (CPR) on scene until return of spontaneous circulation (ROSC) is achieved or until a decision is taken to terminate CPR in physician-staffed emergency medical services (EMS). Transport to a hospital under ongoing CPR is rare in such systems. In selected cases, however, transporting the patient still in cardiac arrest to a hospital for causal treatment of a potentially reversible cause of cardiac arrest is warranted [1, 2]. Some EMS-systems prefer establishing extracorporeal cardiopulmonary resuscitation (eCPR) by implanting a veno-arterial extracorporeal membrane oxygenation (ECMO) on scene [3], whereas other concepts recommend transporting the patient to the hospital under ongoing CPR and establishing ECMO in-hospital [1]. Performing high-quality CPR during transport (consisting of evacuation from the scene to the ambulance, drive to the hospital, and transfer to treatment facility therein) can be challenging. Several aspects have to be considered to decide on the optimal transport modalities, including possibility and potential quality of chest compressions, length and speed of transport, required alterations of patients’ position, required personnel and material, as well as hazards to EMS-staff. In order to deliver high-quality chest compressions and to reduce risks of injury to the personnel during patient transport, mechanical CPR devices can potentially be helpful, even though their use has not proven to be superior to manual CPR and is therefore not recommended for standard CPR in a static situation [4–8]. Additionally, there is a debate about the possibly increased risk of patient injury related to mechanical chest compression devices. To evacuate patients under ongoing CPR from an upper floor, technical and personal assistance from the fire service is usually helpful and close cooperation between EMS and the fire service is crucial. In this study, we sought to establish the optimal strategy for evacuation of a simulated patient under ongoing CPR from the fifth floor. To this end, evacuation via lift, turntable ladder, or staircase was tested, using either manual or mechanical CPR.

## Methods

### Registration and ethical approval

The study was registered in the German Clinical Trials Registry (Deutsches Register für Klinische Studien, www.drks.de, registration number 00012885) and ethical approval was obtained from the institutional ethics committee of the Medical Faculty of the University of Cologne (approval number 17–301).

### Study participants

This study was integrated into the compulsory annual training of paramedics at the fire brigade of the city of Brühl, North Rhine-Westphalia, Germany. The participants were 40 (33 male, 7 female) paramedics of different qualifications (19 *Rettungssanitäter* [lower qualification, similar to EMT in many US states], 17 *Rettungsassistenten* [traditional higher qualification, similar to AEMT in many US states] and 4 *Notfallsanitäter* [new higher qualification, similar to AEMT in many US states] in the German system), between 18 and 60 years of age. They had been informed about the planned scientific evaluation of the training and agreed to participate. No data related to individual participants was collected during the study.

### Experimental setup

Due to the rareness of the scenario in real life and lack of comparability between potential incident scenes, we chose a manikin study design. A CPR-training manikin (Resusci Anne, Laerdal, Stavanger, Norway) was mounted on a spine-board, connected to a defibrillator (Corpuls C3, GS Elektromedizinische Geräte Stemple, Kaufering, Germany) and a respirator (Medumat Standard, Weinmann, Hamburg, Germany). Additional weights were attached to generate a more realistic total weight of 75 kg. Participants were divided into groups of eight (representing two ambulance staff, two emergency physician dispatch car staff, and four fire engine staff). We simulated different evacuation routes and CPR-techniques in a rotating manner, i. e. all participants performed all scenarios during the training day.

**Fig. 1:**
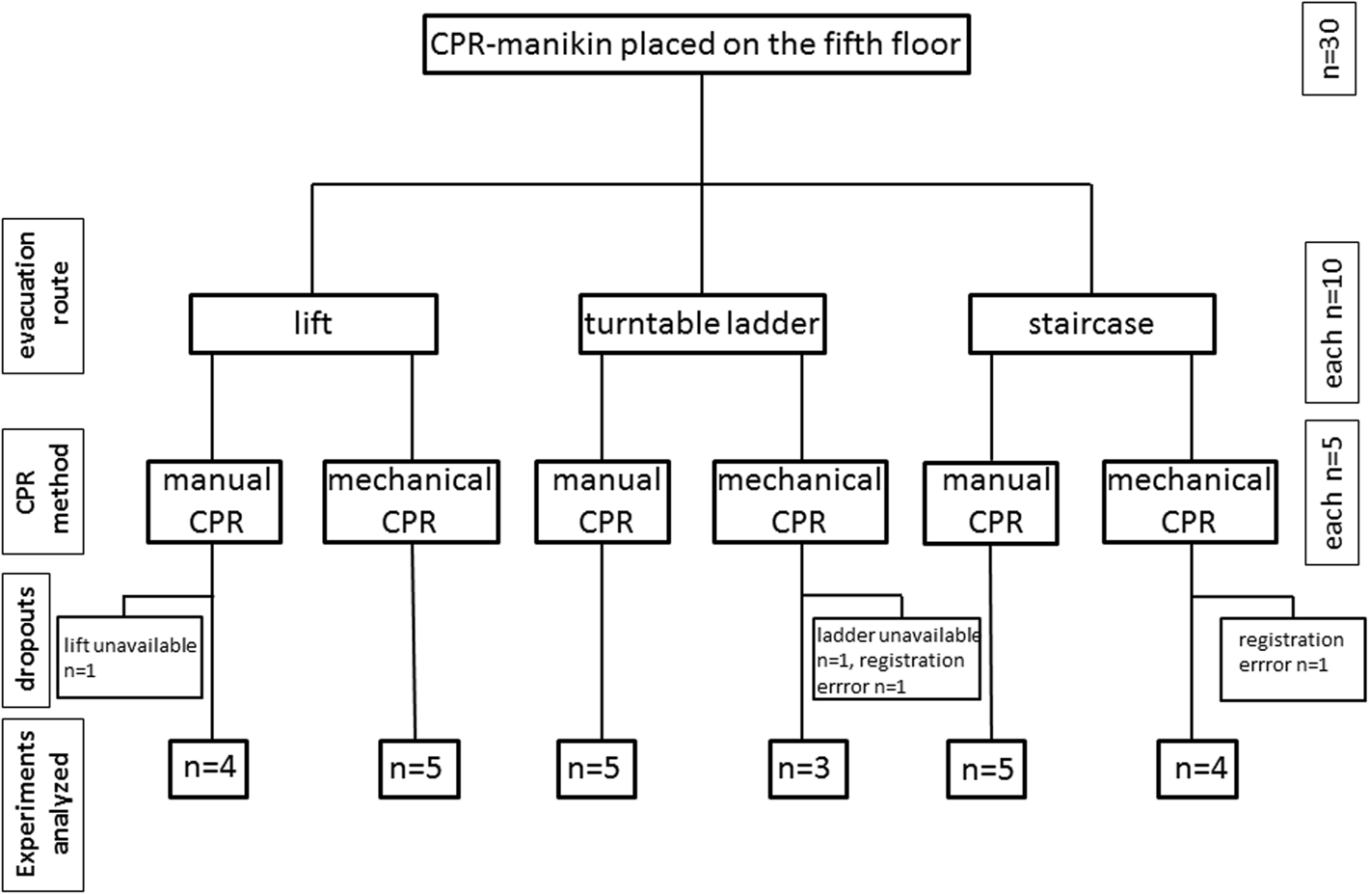
Flowchart of the study setup

To avoid bias due to exhaustion, groups took turns in participation and their roles during resuscitation. Distribution between participants of higher and lower training grades was balanced. Blinding of the participants was not possible. Due to obvious differences in the results observed between manual and mechanical CPR, blinding of data analysts would not have been reasonably possible, either. Each experiment was performed three to five times. The intended number of executions of each scenario was five, there was one dropout in the lift/manual group due to unavailability of the lift, two in the ladder/mechanical group (one due to unavailability of the ladder, one due to a failure of data registration in the SimPad), and one in the stairs/mechanical group due to a failure of data registration in the SimPad (Fig. 1).

In the groups allocated to mechanical CPR, a piston-based CPR-device (Corpuls CPR, GS Elektromedizinische Geräte Stemple) of the EMS of the fire brigade of Brühl was applied. The so-equipped manikin was placed on the floor of an apartment in the fifth floor (UK numbering) of a retirement home. As the aim of our study was to investigate duration and quality of CPR during the evacuation from the building, we measured these parameters from the time of lifting the spine board until arrival at an ambulance parked in front of the building. CPR-events before the start of evacuation, such as endotracheal intubation or attachment of the CPR-device, were not part of the study.

For evacuation via lift, the manikin was lifted on an ambulance stretcher (Stryker M-1, Stryker, Kalamazoo (MI), USA) and transported via a stretcher accessible lift. Participants were free to perform CPR either while walking beside the stretcher or squatting on it. For evacuation via staircase, the spine-board was carried through a large staircase under ongoing CPR. For evacuation via turntable ladder, the upper part of the stretcher was mounted on the stretcher swivel support of the rescue cage of a turntable ladder (DLK 23/12, Magirus, Ulm, Germany) (Additional file 1: photography 1). One firefighter wearing a security harness stayed in the rescue cage and performed chest compressions in the manual CPR group.

### Endpoints of the study

CPR quality was directly measured using a resuscitation training manikin with digital quantification and recording of all CPR measures. Efficiency endpoints were compression depth, frequency of compressions, residual compression of the chest between each compression as a measure of leaning on the chest, and duration of the evacuation. Adverse outomes were disconnection or gross dislocation of the intravenous line, respirator, defibrillator or CPR-device as well as accidents or perceived dangers (subjective measure by the participants and an additional observer) to the personnel during the evacuation.

### Data collection

Duration of evacuation and data on CPR-quality were recorded by a tablet computer (SimPad, Laerdal) connected to the CPR-manikin. Data was extracted from the software provided by the manufacturer (SimView, Laerdal) and transferred to Microsoft Excel for further analysis. Compression depth was measured by the device, registered by the software of the manikin and displayed in 1 mm intervals. Accuracy, according to the manufacturer, is ±15% or 3 mm. Compression frequency was calculated from the time elapsed between two compressions, thus representing a beat-to-beat frequency analysis, not the mean value within a longer (e. g. one minute) time frame. Statistical analysis and graphical layout were performed with Graph Pad Prism 7 (GraphPad Software, San Diego (CA), USA). For comparisons between two groups, the Mann Whitney test was used. For comparison between two independent variables and their interaction, two-way ANOVA was used. A *p*-value of < 0.05was considered statistically significant.

## Results

### Compression depth

**Fig. 2:**
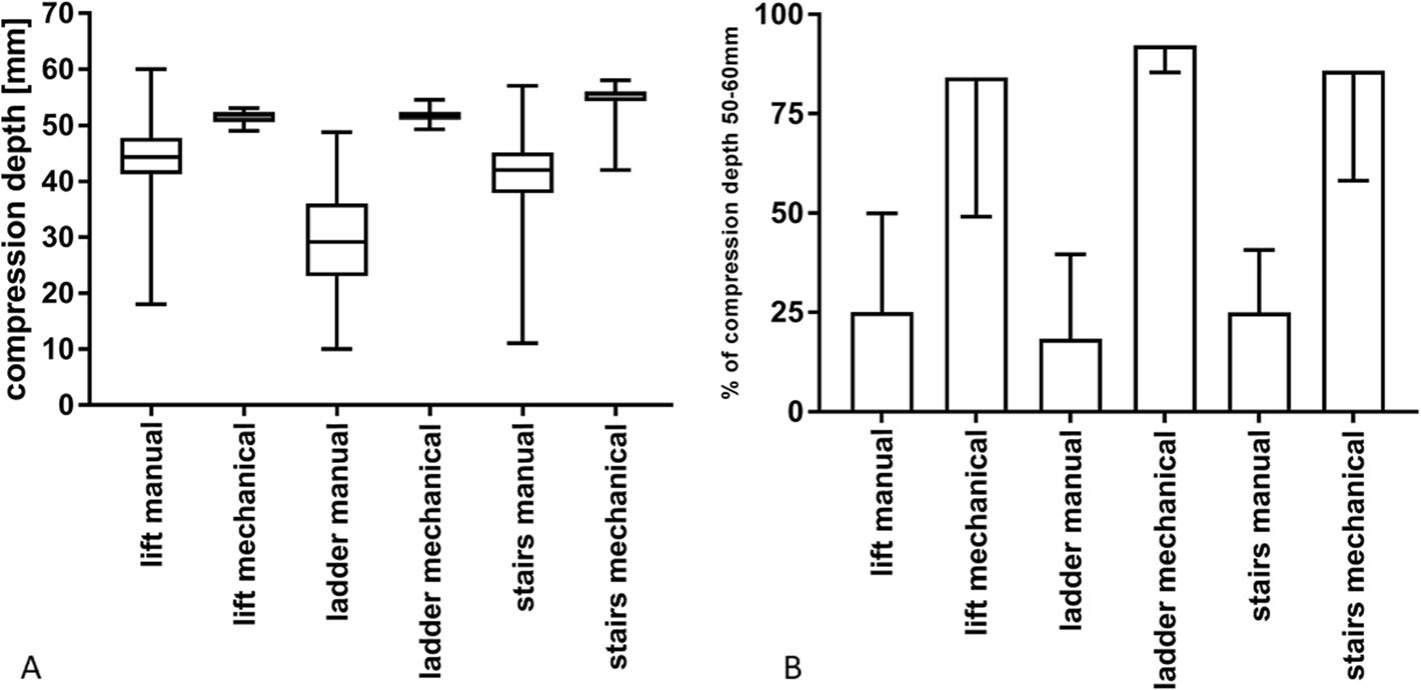
**a**: Compression depth in millimetres. Box whisker plot with minimum, maximum, 25th and 75th percentile and median. **b**: Percentage of guideline-compliant compression depth 50-60 mm. Means and standard deviation (SD)

The compression depth recommended by the guidelines of the ERC is 50 to 60 mm. In our study, compression depth was significantly lower in all manual groups compared with the respective mechanical groups (Fig. 2a, Table 1). Among the manual compression groups, the lowest median compression depth was observed during evacuation via ladder. Especially during the phase of actual movement of the ladder from the fifth floor to the ground, compression depths were low (Additional file 2: Figure S1). The percentage of compression with a depth of 50 to 60 mm, as recommended by current guidelines, was higher with mechanical CPR in all groups (Fig. 2b, Table 1) than with manual CPR, which yielded a lower percentage of correct compression depth. Additionally, in the mechanical groups those compressions not in the target range were almost all very close to its lower end of 50 mm (Additional file 2: Figure S1). Two-way ANOVA showed a statistically significant influence of CPR-method (manual versus mechanical, *p* < 0.001) but not evacuation route (*p* = 0.55) on compression depth, and no interaction between CPR-method and evacuation route (*p* = 0.64).

**Table 1:** Compression depth

Evacuation route	Manual compression	Mechanical compression	*p*-value
lift			
Compression depth (mean ± SD) [mm]	44 ± 5	51 ± 1	< 0.001
% guideline-compliant depth (mean ± SD)	25 ± 25	84 ± 35	0.06
ladder			
Compression depth (mean ± SD) [mm]	30 ± 8	52 ± 1	< 0.001
% guideline-compliant depth (mean ± SD)	18 ± 21	92 ± 7	0.04
stairs			
Compression depth (mean ± SD) [mm]	41 ± 7	55 ± 2	< 0.001
% guideline-compliant depth (mean ± SD)	25 ± 16	86 ± 28	0.02

Means, standard deviation, and *p*-value of compression depth for each evacuation route and CPR-method

### Frequency of compressions

**Fig. 3:**
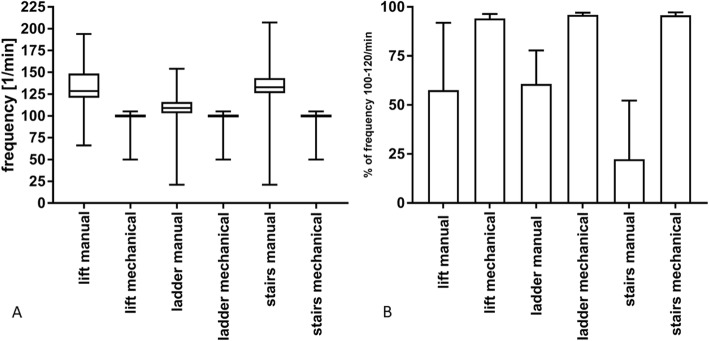
**a**: Compression frequency in 1/min. Box whisker plot with minimum, maximum, 25th and 75th percentile and median. **b**: Percentage of guideline-compliant compression frequency 100–120/min. Means and standard deviation (SD)

The compression frequency recommended by the guidelines of the ERC is 100 to 120 compressions per minute. In our study, wide variations of compression frequency were measured in all manual groups whereas a frequency close to the prescribed 100/min was maintained in all mechanical groups (Fig. 3a, Table 2). The percentage of compressions of a frequency of 100 to 120 per minute, as recommended by current guidelines, was very high in the mechanical groups, mediocre in the lift/manual and ladder/manual, and lowest in the stairs/manual group (Fig. 3b, Table 2). Particularly long hands-off-times were observed in the manual ladder evacuation group during loading the spine-board onto the ladder basket. Here, repetitive interruptions of up to five seconds each occurred (Additional file 2: Figure S1). Two-way ANOVA showed a statistically significant influence of CPR-method (*p* < 0.001) but not evacuation route (*p* = 0.27) on compression frequency, and no interaction between CPR-method and evacuation route (*p* = 0.28).

**Table 2:** Compression frequency

Evacuation route	Manual compression	Mechanical compression	*p*-value
lift			
Compression frequency (mean ± SD) [mm]	133 ± 17	99 ± 4	< 0.001
% guideline-compliant frequency (mean ± SD)	58 ± 34	94 ± 2	0.02
ladder			
Compression frequency (mean ± SD) [mm]	108 ± 14	100 ± 4	< 0.001
% guideline-compliant frequency (mean ± SD)	61 ± 17	96 ± 1	0.04
stairs			
Compression frequency (mean ± SD) [mm]	135 ± 22	100 ± 4	< 0.001
% guideline-compliant frequency (mean ± SD)	22 ± 30	96 ± 2	0.02

Means, standard deviation, and *p*-value of compression frequency for each evacuation route and CPR-method

**Table 3:** Residual compression

Evacuation route	Manual compression	Mechanical compression	*p*-value
lift			
Residual compression (mean ± SD) [mm]	6 ± 3	3 ± 1	< 0.001
ladder			
Residual compression (mean ± SD) [mm]	3 ± 3	1 ± 1	< 0.001
stairs			
Residual compression (mean ± SD) [mm]	2 ± 2	4 ± 1	< 0.001

Means, standard deviation, and *p*-value of residual compression for each evacuation route and CPR-method

### Residual compression between chest compressions / insufficient chest release

**Fig. 4:**
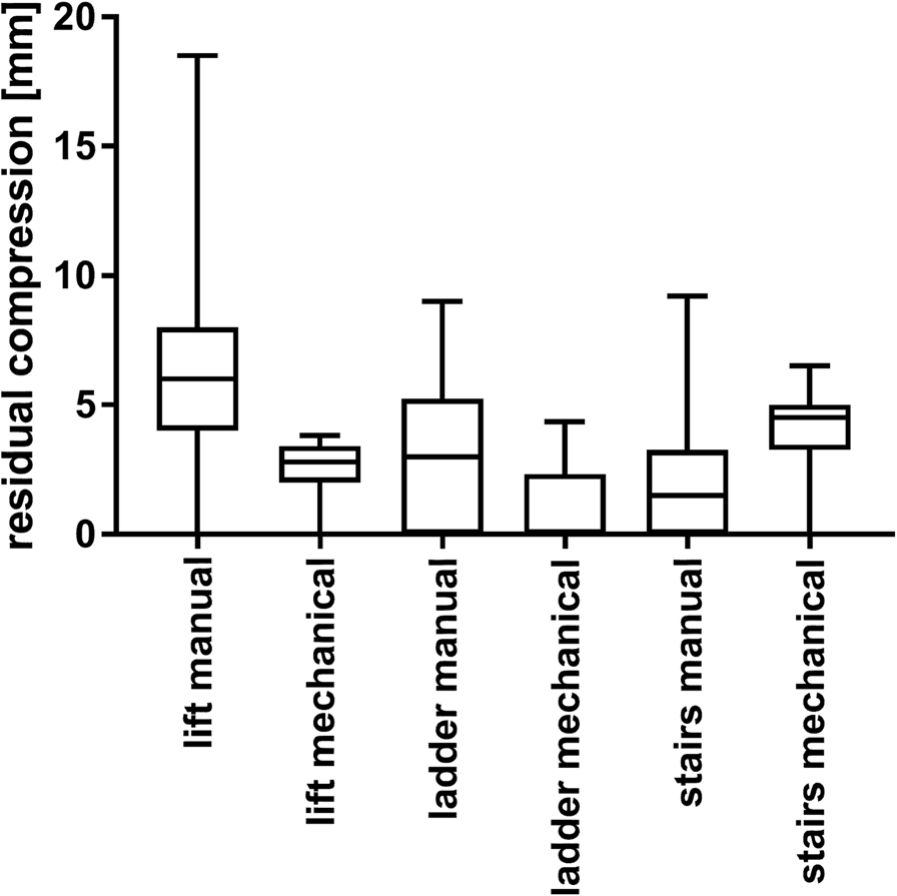
Residual compression of the chest between compressions in millimetres. Means and standard deviation (SD)

The guidelines of the ERC recommend total release of the chest between compressions. In our study, insufficient chest release between compressions varied between groups with a pattern less clear than for compression depth or frequency. For lift and ladder, residual compression of the chest as a measure of insufficient release was more prominent in the manual groups, whereas during evacuation via the staircase, there was more insufficient chest release in the mechanical group (Fig. 4, Table 3). Two-way ANOVA showed no statistically significant influence of CPR-method (*p* = 0.9) or evacuation route (*p* = 0.33) on residual compression, and no interaction between CPR-method and evacuation route (*p* = 0.09).

### Duration

**Fig. 5:**
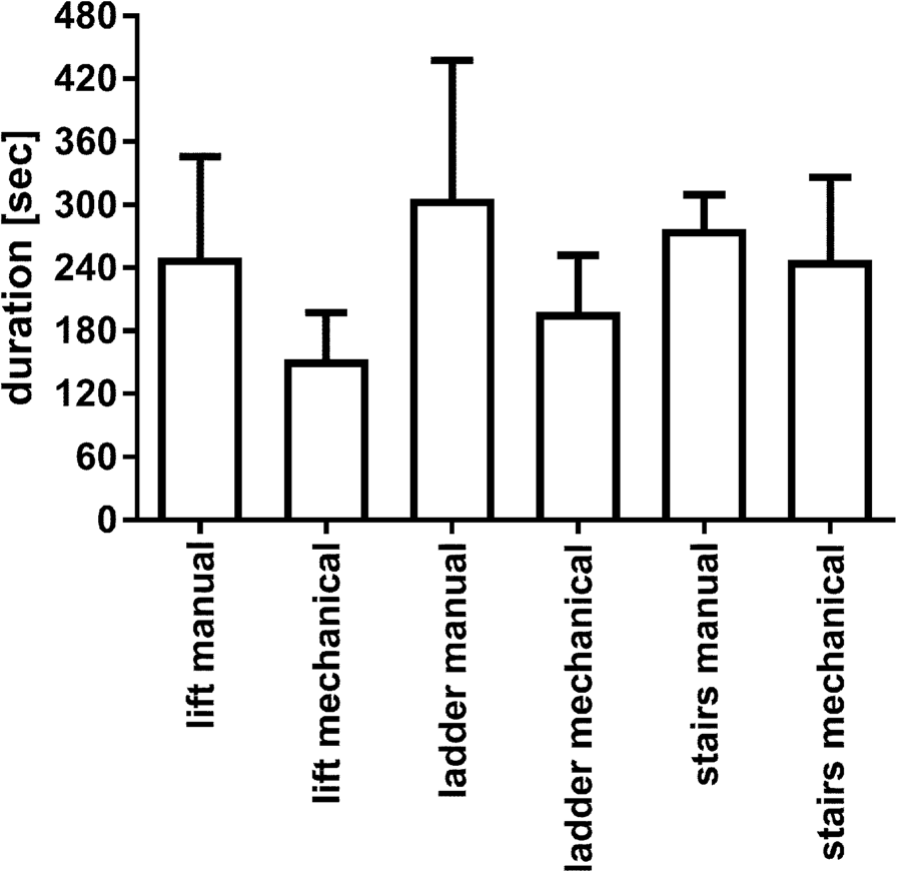
Duration (in seconds) of transport from the fifth floor to the ambulance in front of the building. Means and standard deviation (SD)

Mean duration of transport reached between 152 s in the lift/mechanical group and 306 s in the ladder/manual group. For each evacuation route, evacuation with mechanical CPR was numerically shorter but these differences did not reach statistical significance in the separate analyses of each evacuation route (*p* = 0.127–0.257) (Fig. 5). In the overall analysis of all experiments with two-way ANOVA a statistically significant influence was found for CPR-method (*p* = 0.04), but not for evacuation route (*p* = 0.23) or for interaction between both (*p* = 0.76).

### Adverse outcomes

No disconnections of any device occurred; gross dislocations of the CPR-device were not observed while slight dislocations could not be ruled out by the experimental setup. There were no accidents to the personnel or bystanders. Perceived danger as reported by the participants and the observer to the firefighters performing manual CPR in the turntable ladder basket was high, especially when mounting on the metal bars of the basket to increase efficiency of chest compressions (Additional file 3: photography 2).

## Discussion

We have compared three different evacuation routes and two different CPR-techniques during evacuation under ongoing CPR from a flat in the fifth floor. For all evacuation routes, mechanical chest compressions were found to provide superior CPR-quality in terms of compression depth and frequency. Using a CPR-device, no significant differences in CPR-quality could be detected for evacuation via lift (accessible with a stretcher), turntable ladder, or staircase. For manual chest compressions, particularly poor CPR-quality as well as hazards to the staff were observed during evacuation via turntable ladder. The best results for manual CPR were obtained during evacuation via lift.

Poor manual CPR-quality while moving patients is in accordance with prior publications that studied CPR-quality during other phases of the overall patient transport, e.g. in an ambulance or a helicopter [9–11]. While human and manikin studies focused on “technical” parameters of CPR-quality, such as compression depth and frequency and hands-off-time, a recent trial in a porcine model analysed haemodynamic parameters during manual or mechanical CPR in a moving ambulance. Mechanical resuscitation with the LUCAS-device resulted in higher coronary perfusion pressure, higher end-tidal CO_2_ and lower lactate levels than manual chest compressions [12].

To the best of our knowledge, the phase of transport looked at here – evacuation from a flat in a upper floor to the ambulance – has not been studied in detail yet.

One manikin trial compared CPR-quality with manual or mechanical (LUCAS-device) CPR during transport from a second floor to the hospital, thus including the phase analysed here. The only evacuation route in that study was the staircase. Mechanical CPR was found to result in a higher percentage of compressions with adequate depth (LUCAS 52% vs. manual 36%, *p* < 0.07) and rate (LUCAS 71% vs. manual 40%, *p* < 0.02) during the overall transport. A separate analysis of the phase of evacuation through the staircase is not available [13].

A patient trial analysed manual and mechanical (AutoPulse-device) CPR while moving the patient on a flexible extrication sheet from the site of collapse into the ambulance. No information concerning the sites of collapse is available, so the percentage of patients evacuated from a building, as compared with those evacuated from e. g. a nearby pavement, is unknown. The range of 32 to 540 s for the extrication suggests a diversity of locations. In the manual-CPR-group, chest compressions were interrupted for a median of 270 s versus 39 s in the AutoPulse-group [14].

OHCA in upper floors appears to be related to lower survival rates. Besides demographic or sociological factors, this finding is linked to longer EMS response times. Whether difficulties in evacuation from the upper floors, with or without ongoing CPR, may play a role, cannot be judged from the data provided in the according publications [15, 16]. Our results suggest that in cases of transport under ongoing CPR without a CPR-device, poor CPR-quality has to be expected, possibly contributing to lower survival rates.

From the findings in our study, we suggest using a CPR-device whenever the decision is taken to evacuate a patient under ongoing CPR from an upper floor. If a stretcher accessible lift is available, this route should be preferred, whether CPR be mechanical or manual. Apart from a relatively sufficient CPR-quality for manual CPR, this route avoids tilting of the patient and reduces the need for personnel and material, possibly obviating the need for support from the fire service. If the location of collapse can be accessed with a turntable ladder, this is a suitable route given the availability of a CPR-device. If this is unavailable, CPR-quality during loading and moving of the ladder as well as hazard to the staff is too high to recommend this route. Evacuation via the staircase should be avoided if possible, as it implies tilting of the patient, going along with possible risk of gross dislocation of the CPR-device (which was not observed in our experiments) and strain to personnel (which is required in large numbers). Whether for lower floors than studied here, the practicability of a short staircase transport, compared with bringing a turntable ladder into position, might outweigh these drawbacks, remains undecided from our model of evacuation from the fifth floor.

It remains unclear why chest release between compressions with the Corpuls CPR-device was worse during transport via staircase compared with the other routes. One possible explanation might be slight dislocations of the piston, which is applied with a certain surface pressure at the beginning of treatment, resulting in insufficient release of the chest. Another possible reason might be an oversensitivity of the resuscitation manikin’s pressure sensor.

There is a discussion about the potential of CPR devices to cause musculoskeletal or visceral injuries, such as liver or spleen lacerations. The only randomised trial of device-related injuries so far did not include the Corpuls CPR-device used in this study, but the piston-based device analysed in that study, LUCAS, was found to be non-inferior to manual CPR in terms of severe injuries [17]. By contrast, in two other recently published large studies, patients resuscitated with the LUCAS-device had more resuscitation-related injuries [18, 19], even though this effect lost statistical significance after adjusting for CPR-duration in one of the studies [18]. In case of evacuation from an upper floor, we would still suggest using CPR-devices, even if their injury potential were higher, because of the improvement of CPR-quality versus manual CPR, which performs poorly in this situation.

We recognise three main limitations of this study. Firstly, a manikin study obviously does not permit the analysis of clinical endpoints, such as ROSC, survival or neurological outcome, but had to focus on “technical” parameters of CPR-quality. The low number of real cases, however, precluded the possibility to study this research question in real patients. Secondly, the number of repetitions for each experiment was low. Still, we believe that even with a limited number of repetitions our results are clear enough to support our findings and conclusions in a robust manner. Thirdly, we looked at a clearly defined, isolated phase of transports with all means and personnel readily available at the start of the experiment. In real-life-scenarios, time of attachment of the CPR-device, availability and driving time of the fire service or dimensions of lift or staircase are aspects to be considered. Time intervals such as duration from arrival at the scene to first chest compression or to starting transport to the hospital were not part of this study. Furthermore, participants and investigators were not blinded to the chest compression method and extrication route, which might potentially create a bias. However, CPR-quality during the short stationary phase of the experiment appeared comparable to the CPR-quality observed during general CPR-drills of the fire brigade.

## Conclusions

Our manikin study has shown that mechanical CPR is more effective to perform consistent high quality CPR during evacuation under ongoing CPR from an upper floor. Without mechanical CPR, significant deviations from guideline-recommendations were observed, particularly for evacuation via turntable ladder or stairs. If a stretcher accessible lift is available, this evacuation route offers the possibility of good CPR-quality with low personnel and technical effort. During evacuation via turntable ladder, manual CPR goes along with insufficient CPR-quality and hazards to the staff.

## Supplementary information

**Additional file 1.** Photograph of the manikin fully equipped being loaded onto the turntable ladder.

**Additional file 2.** Representative original tracings of compression depth and frequency from the Laerdal Session Viewer software.

**Additional file 3.** Photograph of a firefighter performing manual CPR in the ladder basket.

## Data Availability

The datasets used and/or analysed during the current study are available from the corresponding author on reasonable request.

## Abbreviations

CPR: Cardiopulmonary resuscitation
ECMO: Extracorporeal membrane oxygenation
eCPR: Extracorporeal cardiopulmonary resuscitation
EMS: Emergency medical services
ERC: European Resucitation Council
OHCA: Out-of-hospital cardiac arrest
ROSC: Return of spontaneous circulation
