# Supplementary figures and images for: Rescue under ongoing CPR from an upper floor: evaluation of three different evacuation routes and mechanical and manual chest compressions: a manikin trial
Source: Scand J Trauma Resusc Emerg Med. 2020 Mar 4;28:16. doi: 10.1186/s13049-020-0709-0 (PMC7055089; doi:10.1186/s13049-020-0709-0)

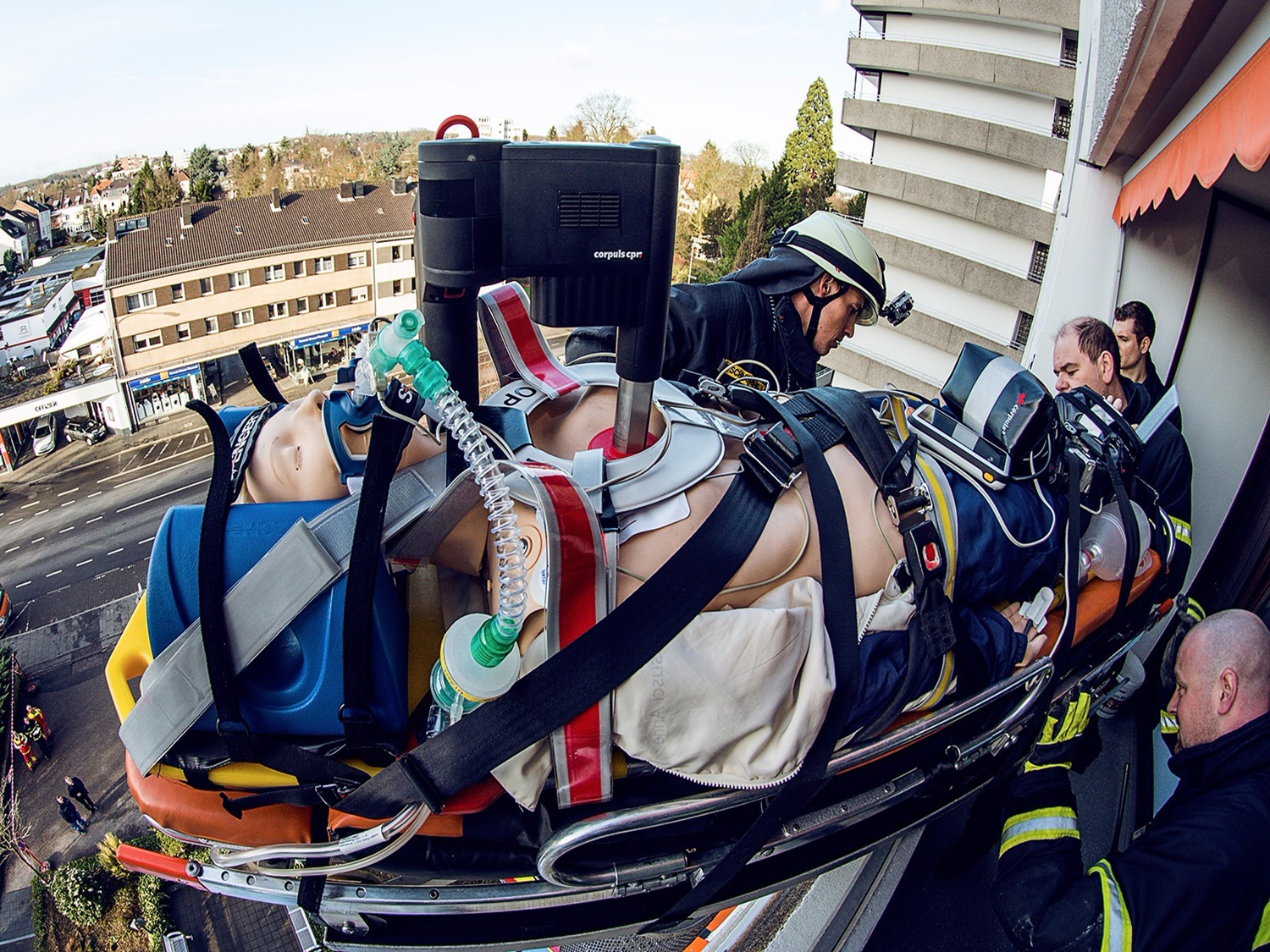

Supplement: Supplementary file 1 — Additional file 1. Photograph of the manikin fully equipped being loaded onto the turntable ladder. [file 13049_2020_709_MOESM1_ESM.tiff]

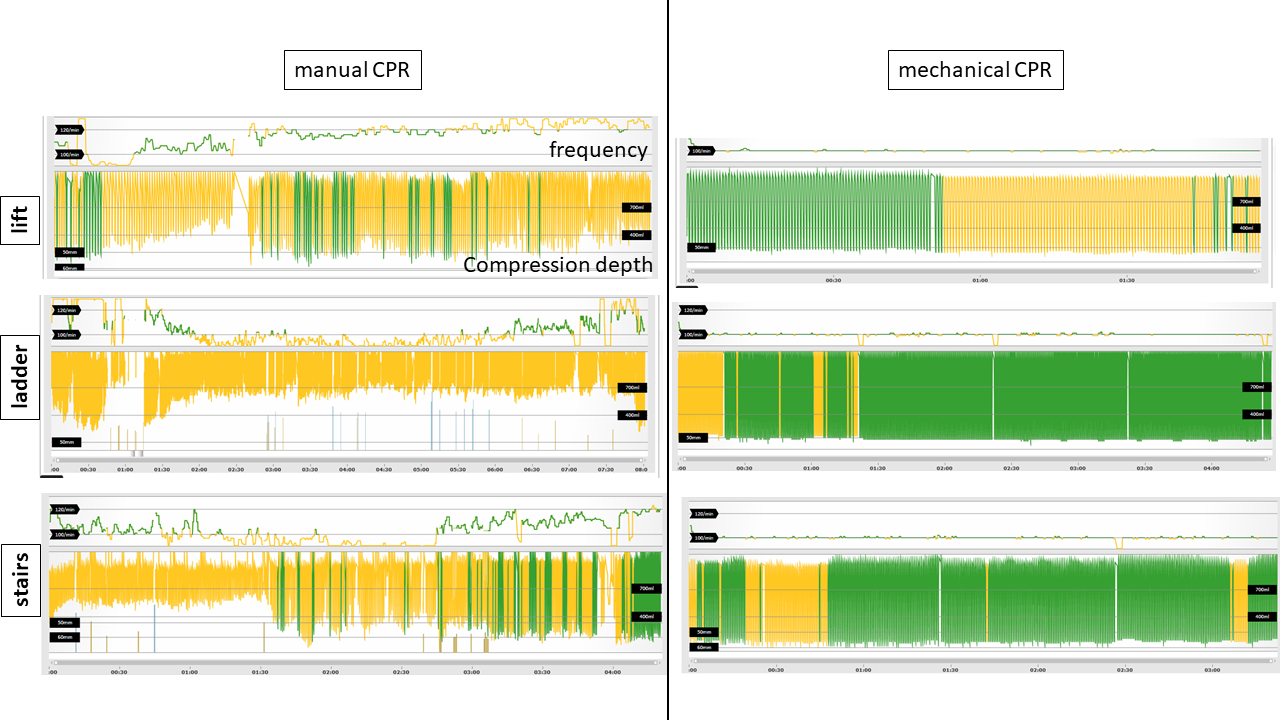

Supplement: Supplementary file 2 — Additional file 2. Representative original tracings of compression depth and frequency from the Laerdal Session Viewer software. [file 13049_2020_709_MOESM2_ESM.tif]

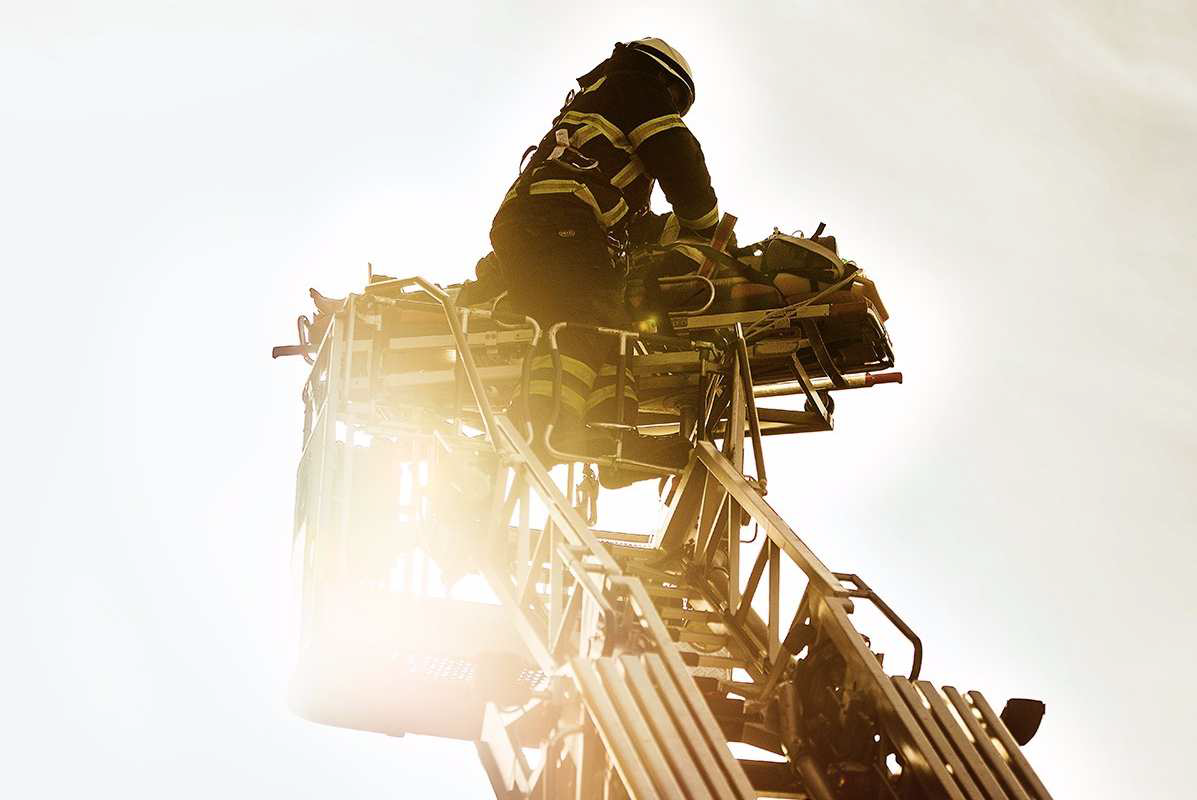

Supplement: Supplementary file 3 — Additional file 3. Photograph of a firefighter performing manual CPR in the ladder basket. [file 13049_2020_709_MOESM3_ESM.tiff]
